# Cervical high-intensity intramedullary lesions in achondroplasia: Aetiology, prevalence and clinical relevance

**DOI:** 10.1007/s00330-012-2488-0

**Published:** 2012-05-26

**Authors:** Patrick A. Brouwer, Charlotte M. Lubout, J. Marc van Dijk, Carmen L. Vleggeert-Lankamp

**Affiliations:** 1 Department of Neurosurgery, Leiden University Medical Centre (LUMC), P.O. Box 9600, NL-2300 RC Leiden, The Netherlands; 2 Department of Neurosurgery, University Medical Center Groningen, Groningen, The Netherlands; 3 Department of Radiology, Leiden University Medical Centre (LUMC), Leiden, The Netherlands

**Keywords:** Achondroplasia, Cervical myelopathy, Congenital spinal abnormality, Dynamic MRI, Medullary compression

## Abstract

**Objectives:**

In achondroplastic patients with slight complaints of medullary compression the cervical spinal cord regularly exhibits an intramedullary (CHII) lesion just below the craniocervical junction with no signs of focal compression on the cord. Currently, the prevalence of the lesion in the general achondroplastic population is studied and its origin is explored.

**Methods:**

Eighteen achondroplastic volunteers with merely no clinical signs of medullary compression were subjected to dynamic magnetic resonance imaging (MRI). The presence of a CHII lesion and craniocervical medullary compression in flexed and retroflexed craniocervical positions was explored. Several morphological characteristics of the craniocervical junction, possibly related to compression on the cord, were assessed.

**Results:**

A CHII lesion was observed in 39% of the subjects and in only one of these was compression at the craniocervical junction present. Consequently, no correlation between the CHII lesion and compression could be established. None of the morphological characteristics demonstrated a correlation with the CHII lesion, except thinning of the cord at the site of the CHII lesion.

**Conclusions:**

CHII lesions are a frequent finding in achondroplasia, and are generally unaccompanied by clinical symptoms or compression on the cord. Further research focusing on the origin of CHII lesions and their clinical implications is warranted.

***Key Points*:**

• *MRI now reveals exquisite detail of the cervical spinal cord.*

• *Cervical cord lesions are observed in one third of the achondroplastic population.*

• *These lesions yield high signal intensity on T2 weighted MRI.*

• *They are generally unaccompanied by clinical symptoms or cord compression.*

• *Their aetiology is unclear and seems to be unrelated to mechanical causes.*

## Introduction

Achondroplasia, with an incidence of 1 in 26,000 live-born infants, is the most common form of dwarfism [1]. Most of these patients have a point mutation in the gene for fibroblast growth factor receptor 3 and more than 80% of these are spontaneous mutations [2–4]. Subsequently, the rate of enchondral ossification is decreased and this leads to a variety of skeletal deformities [2, 5].

Achondroplasia is a form of disproportional dwarfism that not only results in shortened limbs, but also harbours specific distorted morphometrics of the skull and spine. The skull exhibits the characteristic macrocephaly with frontal bossing, saddle-nose deformity, and also a narrow foramen magnum and a short clivus. The achondroplastic spine shows pseudoscalloping of the vertebrae, reduced pedicle length and a reduced interpediculate distance, which all narrow the spinal canal [6]. The deformities of the lower cranium and upper spine predispose patients with achondroplasia to a narrow craniocervical spinal canal, which may lead to cervicomedullary compression.

Previously, we described a remarkable finding in a population of achondroplasts [7]. In a population of patients who visited our neurosurgical clinic for the surgical treatment of neurogenic claudication due to narrowing of the lumbar spinal canal, some patients had concurrent complaints that raised suspicion of spinal cord compression. To that end magnetic resonance (MR) images of the cervical medulla were obtained. Surprisingly, in 64% of those patients a hyperintense lesion in the cervical medulla was observed, without any focal spinal cord compression. We identified these lesions as cervical high-intensity intramedullary lesions (CHII lesions). All CHII lesions typically occurred at a level just below the craniocervical junction, between the arches of C1 and C2, and were confined to the grey matter. The CHII lesions were sometimes associated with local spinal cord thinning, suggestive of focal atrophy of the spinal cord.

The observation was remarkable, as the complaints of these patients were very mild, as opposed to the notable size of the hyperintense lesion in some of these cases. This raised the question of whether this lesion could also be observed in achondroplastic volunteers with no clinical signs of spinal cord compression. Furthermore, interest was raised in the cause of the CHII lesion. It is conceivable that in the neutral position, in which the MR imaging (MRI) was made, no compression at the level of the lesion could be observed, but that it would be present in the flexed or deflexed position of the craniocervical junction. It was hypothesised that flexion or extension of the cranium could further narrow a pre-existing narrow spinal canal, causing transient but repetitive compression of the spinal cord.

The present study is aimed at elucidating the prevalence of the CHII lesion in an otherwise healthy achondroplastic population, and at exploring its aetiology and clinical relevance. Therefore, dynamic MR images of the craniocervical junction in achondroplastic volunteers with no medullary complaints were made, and compression on the upper cervical spinal cord was assessed. Additionally, several aspects of the craniocervical anatomy were correlated to the presence of the CHII lesion (Table 1).

**Table 1:** Characteristics assessed on MRI. Various morphological characteristics of the craniocervical junction that could relate to focal cord compression were assessed. It was investigated whether or not these characteristics correlated with the presence of a CHII lesion

Judging compression	Compression in the AP direction at the craniocervical junction
	Compression in the transverse plane at the level of the CHII lesion
	Atlanto-axial distance in neutral, flexion and retroflexion positions
Craniocervical anatomy	Size of foramen magnum
	Shape of occipital plane
	Angle between clivus and odontoid (clivus spinal cord angle)
	Shape of pons–medulla–cord angle
Tethering of the spinal cord	Is the conus present caudal to L2 and/or are there signs of traction at the medulla in the vertical direction?

## Materials and methods

### Subjects

Achondroplastic participants without neurological symptoms were recruited between August and December 2006 for dynamic MRI of the spine. The Dutch Association of Little People (Belangenvereniging Van Kleine Mensen; www.BVKM.nl) was helpful in contacting volunteers by sending all their members a letter and placing an advertisement on their internet page. Approval of the ethical committee of the of the Leiden University Medical Centre was acquired for this study and written informed consent was obtained from the recruited volunteers.

In order to preclude the volunteers from having symptoms of cervical medullary compression, all recruited participants were scored according to the Myelopathy Disability Index [8] (Table 2), and were only included if their score reached the maximum of 33 points. It was taken into account that the participant’s short stature could complicate the actions described in the Myelopathy Disability Index.

**Table 2:** Myelopathy Disability Index. A validated system, designed by Casey et al. [8], to score the ability of a patient to perform daily household and self-care activities. The score was initially designed for patients suffering from rheumatoid arthritis. All items can be scored as 0 (without any difficulty), 1 (with some difficulty), 2 (with much difficulty) or 3 (unable to do). A maximum score of 33 can be obtained

Score	0	1	2	3
Rising: Are you able to:				
Stand up from an armless straight chair?				
Get in and out of bed?				
Eating: are you able to:				
Cut your meat?				
Lift a full cup or glass to your mouth?				
Walking: are you able to:				
Walk outdoors on a flat surface?				
Climb five steps?				
Hygiene: are you able to				
Wash and dry your entire body?				
Get on and off the toilet?				
Grip: are you able to open jars which have previously been opened?				
Activities: are you able to get in and out of a car?				
Dressing: are you able to dress yourself, including tying shoelaces and buttoning up a shirt or blouse?				
Total score				

### MRI

The MR protocols were tailored to a 1.5-T system (Intera Philips, Eindhoven, The Netherlands). Sagittal T2 TSE SPIR (fat saturated) images of the entire spine were acquired. Sagittal images of the craniocervical junction were obtained in flexed, neutral and deflexed positions using T1 SE and T2 TSE sequences with a surface coil (the flex-m coil, Philips Medical Systems, Best, The Netherlands). Coronal images were obtained in the neutral position using T1 SE and T2 TSE sequences with the same surface coil. Five additional T2-weighted series, using six 0.4-mm-thick midsagittal slices, were acquired with stepwise movement of the head from extreme retroflexion to flexion with the patient in lateral decubitus position. These latter images were used to create dynamic visualisation of the spinal cord movement at the craniocervical junction. Review of the images and measurements were performed using the open-source Osirix DICOM viewer and the OsiriX MD version 1.4 (Pixmeo Sarl, Bernex, Geneva, CH).

### Assessments

The sagittal MR images were independently evaluated for the presence of a CHII lesion by both an experienced neuroradiologist (P.A.B.) and a senior neurosurgeon (C.L.A.V.-L.). In the event of disagreement a consensus reading was performed. A CHII lesion was diagnosed if a typically high-intensity intramedullary signal was seen at the C0–C2 level on T2-weighted sagittal MR images in the neutral position. Also scored was whether there was localised thinning of the spinal cord in the upper cervical region.

Several additional assessments were measured with the use of Osirix® imaging software (version 3.5.2, http://www.osirix-viewer.com) (Table 1).

**Fig. 1:**
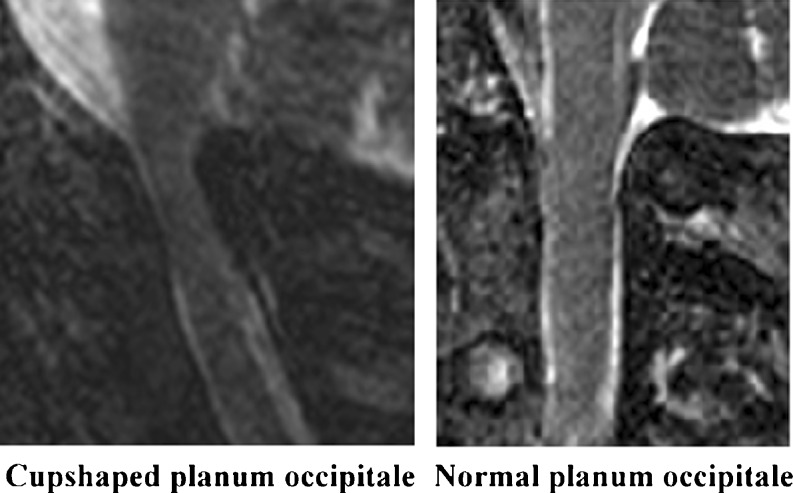
Cup-shaped planum occipitale. In achondroplasia the shape of the occipital plane is often more horizontal than in normal subjects. An occipital plane was considered ‘cup-shaped’ if the opisthion is directed upward in relation to the occipital plane. Typical examples of a cup-shaped and a normally shaped occipital plane are depicted
- The clivus spinal canal angle (CSCA) was assessed by measuring the angle between the Wackenheim–clivus baseline and a line drawn along the posterior aspect of the dens (Fig. 2).

- Compression of the upper cervical spinal cord in AP direction was evaluated on neutral and on flexion/retroflexion MR images. Compression at the level of the CHII lesion in the transverse direction was judged on the coronal images. Compression was scored to be present if there was no cerebrospinal fluid (CSF) visible around the spinal cord (because of a lack of space).
- To evaluate the C1–C2 relation the distance between the posterior surface of the anterior arch of C1 and the anterior surface of the dens (atlanto-dental interval [ADI]) was measured in the neutral position. To evaluate (hyper)mobility of the C1–C2 relation, the ADI was compared in the flexion and extension positions.
- The size of the foramen magnum was registered by measuring the AP diameter from the basion to the opisthion, and the transversal diameter between the lateral curvatures [9]. Also, the size of the spinal canal at the C1–C2 junction (the site of the CHII lesion) in the transverse direction was measured.
- In achondroplasia the shape of the occipital plane is often more horizontal than in normal subjects. We evaluated the shape of the occipital plane and scored this as ‘normal’ or ‘cup-shaped’. An occipital plane was considered ‘cup-shaped’ if the opisthion is directed upward in relation to the occipital plane (Fig. 1).

**Fig. 2:**
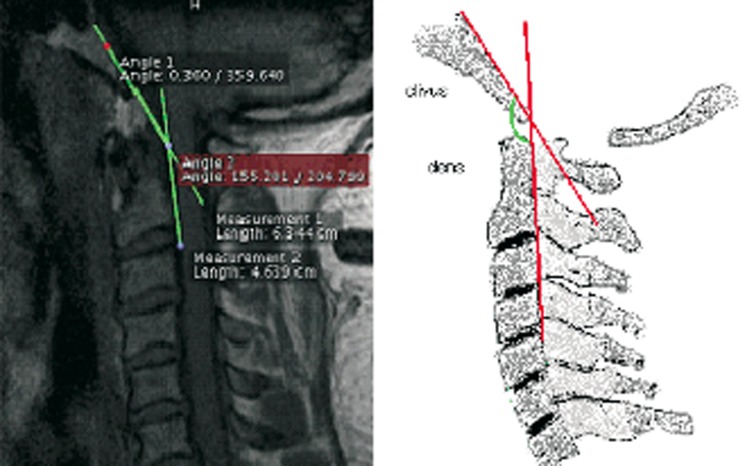
Clivus spinal canal angle. The clivus spinal canal angle is the angle that is formed at the junction of Wackenheim’s line (along the dorsal surface of the clivus) and the posterior vertebral body line. This angle is demonstrated on a subject’s MRI (*left*) and in a drawing (*right*). The clivus canal angle normally ranges from 150° in flexion to 180° in extension, and if this angle is less than 150° the angle is considered to be pathological and may result in spinal cord compression (in the general population) [21]
- The shape of the pons in relation to the spinal cord differed among subjects. Most of the time the pons was slightly bent forward in relation to the cord (anteflexion), but sometimes it was retroflexed (retroflexion) (Fig. 3).

**Fig. 3:**
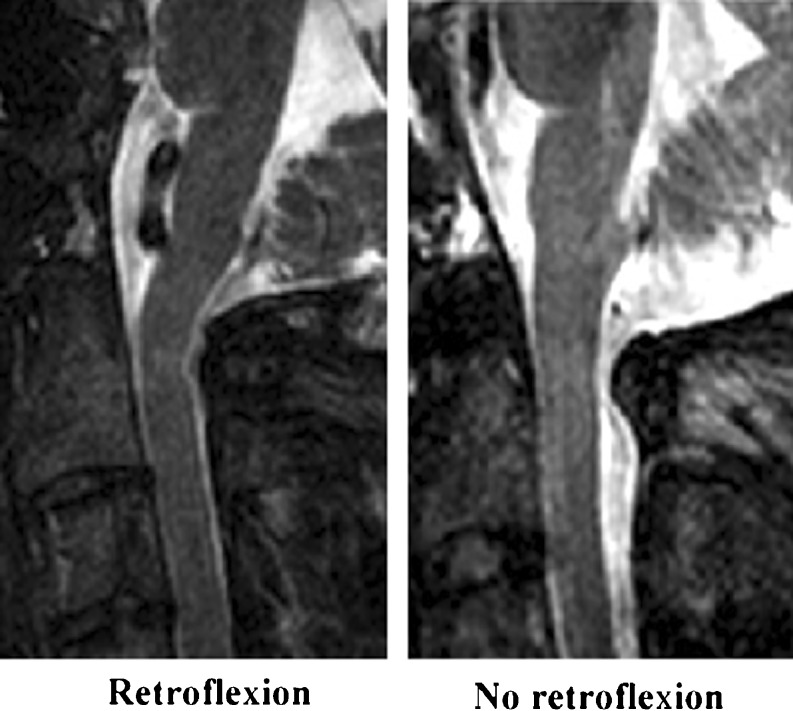
Retroflexion at the pons–spinal cord angle. It appeared that the shape of the pons in relation to the spinal cord also differed between subjects. Most of the time the pons was (slightly) bent forward in relation to the cord (normal), but sometimes it was retroflexed (retroflexion). Four subjects demonstrated a retroflexed shape of the pons–medulla–cord angle, and two of these demonstrated a CHII lesion. Accordingly, there was no correlation with the presence of a CHII lesion (OR 1.80, CI 0.19–17.0)
- Sagittal MR images of the whole spine were screened for tethering of the cord.

### Statistics

The aim of this study was to determine the presence of a correlation between the occurrence of a CHII lesion and one of the (anatomical) variables studied. Categorical data were expressed in frequencies and continuous data were expressed as means ± SD. The correlations between the occurrence of a CHII lesion and the several variables were evaluated with a logistic regression test delivering an odds ratio (OR) with confidence intervals (CI). The degree of concordance among the evaluation of the qualitative measurements was determined by calculating Cohen’s kappa coefficient. Statistical analysis was performed with SPSS 16.0 (SPSS, Chicago, IL, USA).

## Results

Twenty-six achondroplastic subjects volunteered to join the study. All of these patients had a maximum score of 33 on the Myelopathy Disability Index and none had complaints that could in any way be addressed to malfunctioning of the spinal cord. Seven subjects were excluded: two patients were claustrophobic, and five patients declined for personal reasons. Nineteen subjects therefore underwent MRI. One of the subjects appeared to have had a dens fracture in the past and was secondarily excluded.

Three subjects of the population described before [7] volunteered to participate in the present study. These subjects had been surgically treated for neurogenic claudication, and had concurrent complaints that raised suspicion of spinal cord compression. To that end, MR images of the cervical medulla were obtained. At the time of the current MRI study, all complaints had disappeared. This is discussed in more detail below, under the heading “Changes over time in three subjects”. These cases were particularly interesting in order to judge whether the CHII lesion and/or possible compression had changed over time.

All in all, 18 subjects remained in the study. Nine of the subjects were male, nine were female. The mean age was 40.7 ± 15.3 years, ranging from 13 to 64 years.

### Prevalence of the CHII lesion and compression during dynamic MRI

**Fig. 4:**
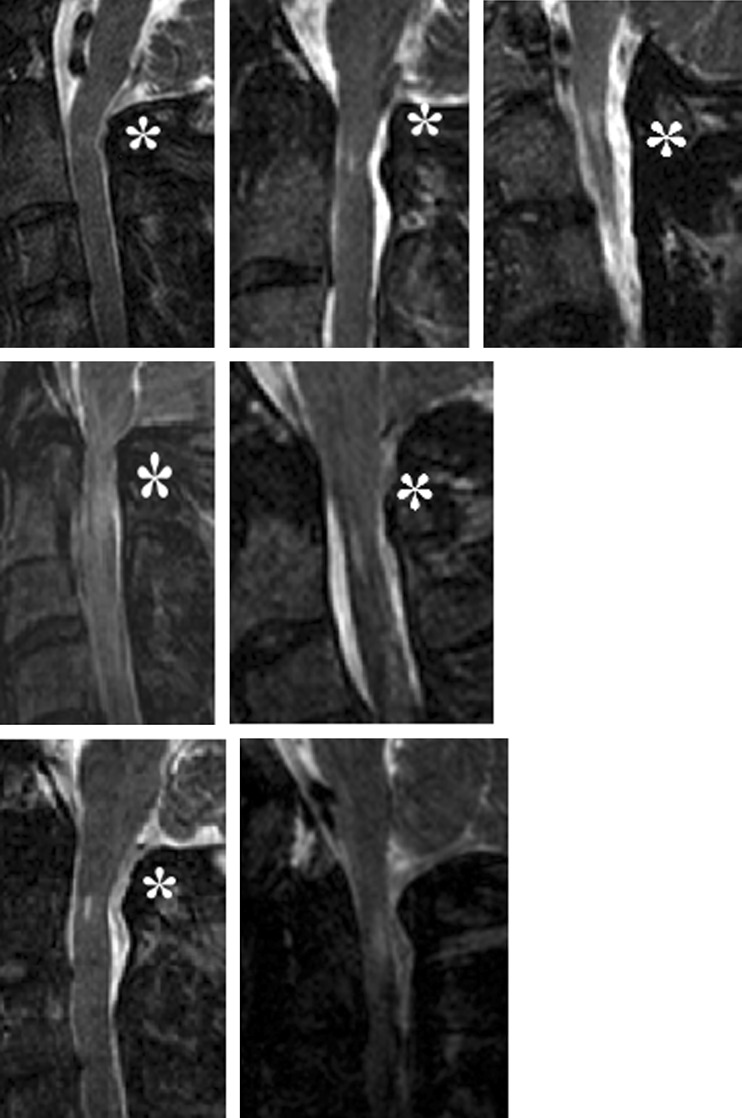
CHII lesions and thinning of the spinal cord. A CHII lesion was diagnosed when a typical high-intensity intramedullary signal was seen at the C0–C2 level on T2-weighted sagittal MR images in neutral position. All subjects demonstrating a CHII lesion are summarised here. Those subjects who demonstrated a CHII lesion and thinning of the spinal cord are marked with an *asterisk*

In 39% of achondroplastic volunteers (seven of the 18 subjects) a CHII lesion was present (Fig. 4; Table 3). In six of them the cervical cord demonstrated thinning of the spinal cord at the spot of the CHII lesion, positively correlating with the presence of a CHII lesion (OR 60.0, CI 3.14–1147) (Fig. 4; Table 4).

**Table 3:** Characteristics. The presence of a CHII lesion, focal cord thinning, compression in the neutral position and compression in the flexed or extended position of the craniocervical spine is summarised

	CHII lesion	Focal cord thinning	Atlanto Dental Interval	Compression in neutral position	Compression in flexed or extended position
1	+	+	2.5	-	-
2	+	+	2.8	-	-
3	+	+	2.2	-	-
4	-	-	2.2	-	-
5	-	-	2.2	-	-
6	-	-	2.4	-	-
7	+	-	2.2	-	-
8	+	+	5.2	Yes	Yes
9	+	+	2.8	-	-
10	-	+	1.7	Yes	Yes
11	-	-	3.0	-	-
12	-	-	2.2	-	-
13	+	+	3.6	-	-
14	-	-	1.7	-	-
15	-	-	2.2	-	-
16	-	-	2.2	-	-
17	-	-	3.3	-	-
18	-	-	3.3	-	-

**Table 4:** Logistic regression and cross-tab statistics. The presence of a CHII lesion was correlated with age, foramen magnum anatomy, clivus spinal canal angle, transverse diameter of the canal at the C1–C2 facet joint, gender, compression on the cord, thinning of the cord, shape of the pons–spinal cord angle and shape of the occipital plane

Continuous variable	Odds ratio	Confidence interval
CHII – age	0.953	0.88–1.02
CHII – for magn AP	0.08	0.03–2.00
CHII – for magn transv	0.70	0.01–80.06
CHII – for magn APxtransv	0.51	0.10–2.74
CHII – CSCA neutral	0.96	0.84–1.11
CHII – CSCA flex	1.01	0.86–1.19
CHII – CSCA ext	1.02	0.90–1.15
CHII – transv diam C1–C2	0.85	0.59–1.22
Binary variable		
CHII – compression	1.67	0.09–31.9
CHII – gender	0.23	0.03–1.77
CHII – thinning cord	60.0	3.14–1147
CHII – shape pons-cord	1.80	0.19–17.0
CHII – planum occipitale	1.80	0.19–17.0

**Fig. 5:**
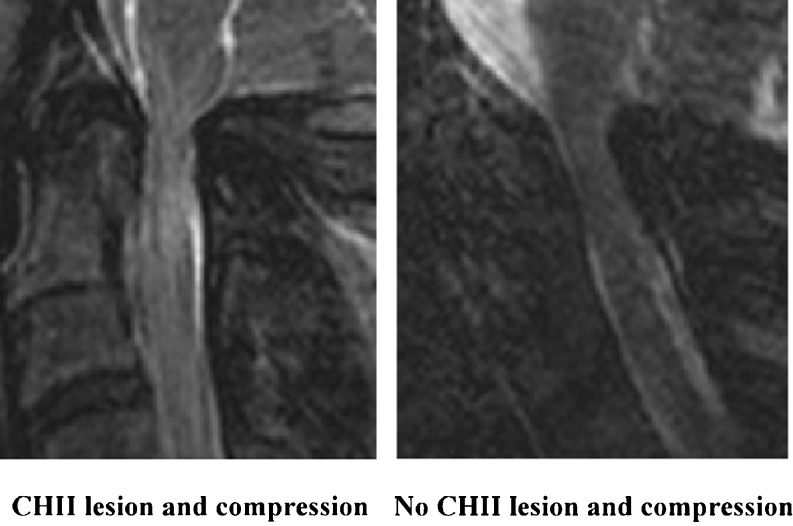
Compression of the spinal cord at the craniocervical junction. MR images of the two subjects who exhibited compression on the spinal cord at the craniocervical junction in the neutral position

The flexion and extension MR images demonstrated that subjects could indeed flex and retroflex their heads sufficiently. In two subjects there was AP compression on the spinal cord in the neutral position (Fig. 5), without a change upon flexion or retroflexion. One of these subjects had a CHII lesion, while the other had not. None of the patients demonstrated compression on the spinal cord in the transverse direction. The dynamic MR images showed no transitory compression. Consequently, no correlation between the presence of a CHII lesion and compression on the cervical spinal cord could be established (OR 1.67, CI 0.09–31.9). Nor did the CHII lesion correlate with age (OR 0.953, CI 0.88–1.02) or gender (OR 0.23, CI 0.03–1.77).

Comparison of the data from this study with those of our previous study [7] showed no statistical difference in the occurrence of the CHII lesion between the symptom-free subjects and the group with minimal complaints (Fisher’s exact test *P* = 0.130).

### Morphometric characteristics

The morphometric characteristics are summarised in Tables 3 and 5, and the correlations are summarised in Table 4. Data on the ADI are summarised in Table 6. None of the subjects demonstrated tethering of the cord.

**Table 5:** Morphological characteristics. The size of the foramen magnum, the shape of the planum occipitale, the clivus–spinal canal angle in the flexion and extension position, and the shape of the pons–medulla–cord angle are summarised. The presence of a CHII lesion is indicated. The size of the foramen magnum reported in the general population is 29 × 34–35 mm [20, 21]. The transverse diameter is thus halved in achondroplasts

	Foramen magnum AP	Foramen magnum transversal	Planum occipitale	Clivus spinal canal angle (flex-ext) in degrees	Shape craniocervical cord	CHII lesion
1	30.1	12.3	Normal	172-177	Normal	+
2	28.0	17.0	Normal	168-159	Retroflexion	+
3	27.2	13.6	Cup-shaped	166-168	Normal	+
4	29.6	12.0	Normal	167-158	Normal	-
5	32.9	11.5	Normal	161-167	Normal	-
6	32.1	14.6	Normal	171-178	Normal	-
7	32.0	13.0	Normal	168-177	Normal	+
8	20.0	n.a.	Cup-shaped	172-173	Retroflexion	+
9	26.5	n.a.	Normal	155-151	Normal	+
10	30.0	19.0	Cup-shaped	168-163	Retroflexion	-
11	25.4	13.0	Normal	175-175	Normal	-
12	27.4	16.7	Normal	160-155	Normal	-
13	25.1	13.7	Normal	176-176	Normal	+
14	30.5	11.7	Normal	174-175	Normal	-
15	26.9	13.6	Normal	167-168	Normal	-
16	36.0	11.0	Normal	174-171	Normal	-
17	26.0	17.0	Cup-shaped	159-164	Retroflexion	-
18	39.0	15.0	Normal	170-168	Normal	-

**Table 6:** Morphological characteristics and correlates of the atlanto-dental interval (ADI). An atlanto-dental interval on CT in adults is considered normal if it is less than 3 mm [22], but it may be accepted up to 5 mm if the difference between the ADI in flexion and extension does not exceed 5 mm. These borders are derived from the literature on patients suffering from rheumatoid arthritis and it is doubtful whether these values should be strictly extrapolated to the achondroplastic population. The atlanto-dental interval data correlated with the CHII lesion, and no correlation could be established. There was one patient with an ADI of 5.2 mm (Table 3, number 8), an ADI flexion of 3.8 mm, and an ADI extension of 2.5 mm. This patient did demonstrate compression of the craniocervical junction, had a cup-shaped occipital plane, had a retroflexed dens and thinning of the medulla at the craniocervical junction, and a CHII lesion

	Range (mm)	Median (mm)	Correlation with CHII lesion
ADI neutral	1.7–5.2	2.3	OR 1.13 (CI 0.96–1.33)
ADI flexion	1.4–4.4	3.3	OR 182.8 (CI 0.0–8633182)
ADI extension	1.2–4.2	2.7	OR 0.44 (CI 0.0–232604)
Difference in ADI flexion/extension	0–2.5	0.7	OR 1.00 (CI 0.96–1.05)
ADI > 3 mm			OR 1.8 (CI 0.19–17.0)

The interobserver variabilities indicate substantial agreement for the compression in neutral position and focal cord thinning, and almost perfect agreement [10] for the other qualitative parameters (Table 7).

**Table 7:** Kappa interobserver variability. Qualitative data were judged by a neuroradiologist and a neurosurgeon. Cohen’s Kappa interobserver coefficient was calculated

	Kappa interobserver variability
CHII lesion in neutral position	0.88 ± 0.23
Compression in neutral position	0.64 ± 0.22
Compression in flexion/extension	1.00
Focal cord thinning	0.75 ± 0.24
Planum occipitale	0.82 ± 0.23
Shape of the craniocervical cord	0.82 ± 0.23

### Changes over time in three subjects

Three subjects were both in the previous study [7] and the current study: numbers 7, 10 and 14. In subject number 7 (age 13), the first MRI was performed because the patient complained of paroxysmal paraesthesiae of his arms and hands. The ‘attacks’ disappeared after a few months. The CHII lesion was present on the first MRI and also on the MRI that was made for the current study. There was no compression on the cord, and nothing had changed after 17 months.

Subject number 10 (age 56) complained initially about his legs and urination. The MRI revealed compression at the craniocervical junction and on the medulla at a thoracic level. No CHII lesion was present. After decompression of the medulla at the thoracic level, the patient was free of symptoms. Therefore the craniocervical junction was not operated on. At the time of the current MRI, the patient did not have any complaints, although the compression at the craniocervical junction was comparable with the MRI that had been made 7 years earlier. There was still no CHII lesion.

Subject number 14 (age 62) was initially subjected to an MRI because of paroxysmal subtle sensibility changes in the skin of the abdomen and back. The MRI did not demonstrate a CHII lesion or compression of the cord. The complaints disappeared spontaneously after a few months. The current MRI still demonstrated no CHII lesion. In conclusion, these three subjects were free of neurological symptoms at the time of recruitment and the MR images were unchanged over time.

## Discussion

This study demonstrates that about 40% of the achondroplastic population exhibit a hyperintense lesion in the upper cervical spinal cord (CHII lesion). The presence of this lesion is unaccompanied by clinical symptoms, and there is no focal compression on the cord in the neutral, the flexed or retroflexed positions of the craniocervical junction. There is no indication for instability of the upper cervical spinal column in any of the subjects, as the ADI was within the normal range in all subjects. Moreover, no correlation could be established between the CHII lesion and the size of the ADI or the difference in ADI upon flexion and retroflexion.

In observing the size of the foramen magnum of the subjects (Table 5) it was obvious that the distance in the transverse direction was less than half the distance in the anteroposterior direction. This is due to a smaller interpedicular distance in achondroplastic subjects. We hypothesised that judging compression on the cord in the sagittal plane, as we are used to in non-achondroplastic subjects, is not optimal for achondroplasts, as the size of the canal is limiting in the smaller coronal plane. Therefore, we also judged the coronal images and measured the size of the canal, not only at the foramen magnum, but also at the site of the CHII lesion. However, no compression was observed in the coronal plane either, and no correlation could be established between the CHII lesion and size of the canal, neither at the craniocervical junction, nor at the site of the CHII lesion.

The histopathological correlate of the CHII lesion is unknown. Oedema is not likely the cause of the CHII lesion, as there was thinning rather than swelling of the cord. It is therefore most probable that the CHII lesion represents gliosis. A subsequent study in which achondroplastic subjects with a CHII lesion will undergo MR spectroscopy imaging may reveal the true nature of this lesion [11].

Although no correlation between the presence of a CHII lesion and cord compression could be demonstrated at the time point at which the subjects were investigated, it is imaginable that the subjects demonstrating a CHII lesion were subjected to craniocervical compression on the cord in early childhood. The combination of macrocephaly with the marked hypotonia and the poor head control noted in infants with achondroplasia is prone to cause the head to overflex and/or over-retroflex. In combination with other anatomical characteristics of achondroplasia, such as a small foramen magnum and a narrow spinal canal, repetitive compression at the craniocervical junction is a possible explanation. It has been suggested before that impingement on the cord in early childhood may return to normal upon maturation [12].

Another possibility is that the lesion is not caused by direct compression of anatomical structures, but that the craniocervical junction is so narrow in childhood that it causes a venous outflow problem with minimal repetitive intramedullary damage as a result. Bruhl et al. [13] described a patient with a syrinx-like structure reaching from rostral to caudal to the craniocervical junction in combination with severe compression at the rim of the foramen magnum. After decompressive surgery had been performed, a small hypointense lesion, like a CHII lesion, remained present. Bruhl et al. related the syrinx-like structure to a venous outflow deficiency due to the craniocervical compression. The CHII lesions that we described in this article may be the remnants of syrinx-like structures that diminished upon maturation and thus increased the size of the foramen magnum.

Sudden infant death is described to occur in up to 7.5% of achondroplastic children [14, 15], without a satisfactory explanation. Therefore, interest is raised in phenomena that may reveal the cause of this. The presence of a CHII lesion raises suspicion that compression of the cord was present during early childhood. Compression on the cord at the craniocervical junction has been demonstrated before in achondroplastic children [16], which sometimes could only be revealed upon flexion and/or retroflexion [17]. In these patients, however, no hyperintense lesions in the cervical medulla on T2-weighted MR images were reported, although they did demonstrate clinical symptoms that could be related to craniocervical cord compression like (mild) hypotonia and nocturnal apnoea. Unfortunately, the achondroplastic features make it sometimes difficult to discern symptoms pointing in the direction of cord compression from symptoms based on upper airway problems. Achondroplasts regularly suffer from obstructive apnoea attributable to upper airway causes such as mid-facial hypoplasia and adeno-tonsillar hypertrophy [18, 19]. It may be hard to discern this obstructive apnoea from central apnoea because of compression on the cord, but polysomnography may be helpful in this. It can also be difficult to judge whether a (mild) tetraparesis is pathological and due to compression on the cord, or physiological, as the achondroplastic child is well known to be hypotonic during early infancy, and to achieve motor milestones at a slightly slower pace than unaffected individuals [20].

The absence of clinical symptoms in patients exhibiting a CHII lesion can be a matter of debate. In the current study, all volunteers had a maximum Myelopathy Disability Index score, which is a rather coarse measure and the presence of minimal complaints or symptoms cannot be ruled out. Perhaps it is particularly a group of subjects who experienced some awkward sensations who were willing to volunteer to participate in this study. It can be concluded that the often impressive CHII lesion is remarkably unaccompanied by significant complaints, but the occurrence of slight symptoms cannot be ruled out.

As it seems today, with the current knowledge of the CHII lesion, the possession of this lesion, without concurrent compression or symptoms, has no clinical consequence. It does not warrant further diagnostic imaging or intervention. Our study is limited by the restricted number of patients examined in this study and the experimental nature of the dynamic MRI. The prevalence of achondroplasia is low and it is therefore difficult to gather a large number of patients. In comparison to the literature available, the current study describes a fair number of patients. The dynamic MRI technique used in the present study has to our knowledge not been described to date, and validation of this technique would be of value.

In conclusion, CHII lesions are a frequent finding in the achondroplastic population, and are remarkably merely unaccompanied by clinical symptoms. No correlation with static or dynamic compression of the spinal cord could be established. In the absence of symptoms, the presence of a CHII lesion is not dangerous and regular follow-up is not obligatory. The aetiology of CHII lesions remains unknown and further research focusing on their origin is warranted.

## References

[1] Oberklaid F, Danks DM, Jensen F, Stace L, Rosshandler S (1979). Achondroplasia and hypochondroplasia. Comments on frequency, mutation rate, and radiological features in skull and spine. J Med Genet.

[2] Horton WA, Hall JG, Hecht JT (2007). Achondroplasia. Lancet.

[3] Yamanaka Y, Ueda K, Seino Y, Tanaka H (2003). Molecular basis for the treatment of achondroplasia. Horm Res.

[4] Murdoch JL, Walker BA, Hall JG, Abbey H, Smith KK, McKusick VA (1970). Achondroplasia—a genetic and statistical survey. Ann Hum Genet.

[5] Maroteaux P, Lamy M (1964). Achondroplasia in man and animals. Clin Orthop Relat Res.

[6] Epstein JA, Malis LI (1955). Compression of spinal cord and cauda equina in achondroplastic dwarfs. Neurol.

[7] van Dijk JM, Lubout CM, Brouwer PA (2007). Cervical high-intensity intramedullary lesions without spinal cord compression in achondroplasia. J Neurosurg Spine.

[8] Casey AT, Bland JM, Crockard HA (1996). Development of a functional scoring system for rheumatoid arthritis patients with cervical myelopathy. Ann Rheum Dis.

[9] Aydin S, Hanimoglu H, Tanriverdi T, Yentur E, Kaynar MY (2005). Chiari type I malformations in adults: a morphometric analysis of the posterior cranial fossa. Surg Neurol.

[10] Landis JR, Koch GG (1977). The measurement of observer agreement for categorical data. Biom.

[11] Denic A, Bieber A, Warrington A, Mishra PK, Macura S, Rodriguez M (2009). Brainstem 1H nuclear magnetic resonance (NMR) spectroscopy: marker of demyelination and repair in spinal cord. Ann Neurol.

[12] Rimoin DL (1995). Cervicomedullary junction compression in infants with achondroplasia: when to perform neurosurgical decompression. Am J Hum Genet.

[13] Bruhl K, Stoeter P, Wietek B, Schwarz M, Humpl T, Schumacher R (2001). Cerebral spinal fluid flow, venous drainage and spinal cord compression in achondroplastic children: impact of magnetic resonance findings for decompressive surgery at the cranio-cervical junction. Eur J Pediatr.

[14] Hecht JT, Francomano CA, Horton WA, Annegers JF (1987). Mortality in achondroplasia. Am J Hum Genet.

[15] Pauli RM, Scott CI, Wassman ER, Gilbert EF, Leavitt LA, Ver Hoeve J (1984). Apnea and sudden unexpected death in infants with achondroplasia. J Pediatr.

[16] Bagley CA, Pindrik JA, Bookland MJ, Camara-Quintana JQ, Carson BS (2006). Cervicomedullary decompression for foramen magnum stenosis in achondroplasia. J Neurosurg.

[17] Danielpour M, Wilcox WR, Alanay Y, Pressman BD, Rimoin DL (2007). Dynamic cervicomedullary cord compression and alterations in cerebrospinal fluid dynamics in children with achondroplasia. Report of four cases. J Neurosurg.

[18] Ottonello G, Villa G, Moscatelli A, Diana MC, Pavanello M (2007). Noninvasive ventilation in a child affected by achondroplasia respiratory difficulty syndrome. Paediatr Anaesth.

[19] Collins WO, Choi SS (2007). Otolaryngologic manifestations of achondroplasia. Arch Otolaryng Head Neck Surg.

[20] Pauli RM, Horton VK, Glinski LP, Reiser CA (1995). Prospective assessment of risks for cervicomedullary-junction compression in infants with achondroplasia. Am J Hum Genet.

[21] Smoker WR, Khanna G (2008). Imaging the craniocervical junction. Childs Nerv Syst.

[22] Khanna G, Sato Y (2005). Imaging of the craniovertebral junction. Oper Techn Neurosurg.

